# How general functioning of family affects gambling-related beliefs: the mediating role of communication and the moderating role of impulsivity trait

**DOI:** 10.3389/fpsyt.2023.1165053

**Published:** 2023-07-13

**Authors:** Dapeng Zhang, Shuang Zhou, Hui Zheng, Lei Guo, Jing Zhai, Ziqi Liu, Zheyi Du, Ping Dong, Min Zhao, Jiang Du

**Affiliations:** ^1^The Third People's Hospital of Fuyang, Fuyang, China; ^2^Shanghai Mental Health Center, Shanghai, China; ^3^Shanghai Key Laboratory of Psychotic Disorders, Shanghai Jiao Tong University School of Medicine, Shanghai, China; ^4^Center for Excellence in Brain Science and Intelligence Technology, Chinese Academy of Sciences, Shanghai, China

**Keywords:** family functioning, gambling disorder, parent-child communication, impulsivity, moderation mediation model

## Abstract

**Background:**

Gambling behaviors can be exhibited by individuals raised in families with impaired parent-child communication and individuals with more impulsive traits. However, it remains unclear how gambling-related beliefs are modulated by impulsivity traits and parent-child communication styles.

**Methods:**

A total of 95 adult patients (age ≥ 18 years) diagnosed via DSM-5 criteria with gambling disorder (GD) completed our questionnaire. Participants filled out pen-and-paper questionnaires that included basic demographic information, the Family Assessment Device (FAD), Parent-Adolescent Communication Scale (PACS), Gambling Attitude and Belief Survey (GABS), and Barratt Impulsiveness Scale (BIS). We used a moderation mediation model to explore the relationship between variables. The study results were considered statistically significant if *p* < 0.05, or the 95% confidence interval did not contain zero.

**Results:**

The scores of the problems in communication with mother subscale (PCMS) of PACS were significantly positively correlated with the scores of GABS and the general functioning 12-item subscale (GF12) of FAD. The relationship between the scores of GF12 and GABS was completely mediated [β = 4.83, (1.12, 10.02)] by PCMS scores, and the BIS scores moderated this relationship: the predictive path between GF12 and PCMS scores [index of moderated was β = −0.25, (−0.60, −0.04)], and the indirect predictive front path between the scores of GF12 and GABS were significant only in subjects with low BIS scores.

**Conclusion:**

These findings suggest that poor general functioning of the family may increase gambling-related beliefs as a result of communication problems with mothers, and this result is only significant for individuals with low impulsivity. When treating patients with GD, more treatment of mother-child communication issues and individual impulsivity may be more conducive to their recovery.

## 1. Introduction

Gambling disorder (GD) is defined as persistent pathological gambling behavior driven by distorted gambling beliefs, and it is considered to be associated with a significant impairment in family and occupational functioning, estrangement of parent-child relationships, and impulsive personality traits (1–3). According to the theory of Developmental Psychopathology and Family Process (4), we believe that risk factors in the family environment are one of the decisive factors for the occurrence and development of children's aberrant cognition, belief, and behavior. According to Bayes' Theorem, pathological behavior, such as addictive behavior, is guided by abnormal prior beliefs (5). Gambling-related beliefs have been shown to predict gambling behavior (6). Gambling behaviors can be exhibited by individuals raised in families with impaired parent-child communication and by individuals who are more impulsive (7–9). However, it remains unclear how gambling-related beliefs are influenced by family functioning and modulated by impulsive traits and parent-child communication styles.

Researchers believe that family functioning is a protective factor for gambling beliefs (10). Family functioning is the ability of the family as a whole to meet the various needs (such as emotional communication) of family members (11). As an important factor influencing health behaviors (12), it is related to a variety of adolescent risk behaviors. The family shapes the attitudes, beliefs, and norms that influence the children's behaviors as they transition from adolescence to adulthood. In one cross-cultural study, it was discovered that family functioning was not a direct significant predictor of at-risk/pathological gambling (13). Other studies have found that problem gamblers seeking treatment report greater family dysfunction (9, 14, 15). These inconsistencies may indicate that family functioning itself may not be sufficient or a direct predictor of gambling attitudes, beliefs, and behaviors.

Parent-child communication, an interactive process based on the parent-child relationship, is a bridge between family functioning and several unhealthy gambling-related beliefs. Family systems theory is used to guide our examination of the influence of subsystems (i.e., the parents, their communication, monitoring, and parenting styles) on the children's gambling-related beliefs because it focuses on the interaction processes between parents and the child (16). The study by Maggie and Ingersoll found that the lower the level of trust and communication between children and their parents, the more likely they are to become problem gamblers (17). Additionally, Lei et al.'s study found that the general functioning of a family only affects communication between mother and child (18). Some studies from China suggest that mother-child have more common interactions and communication than father-child (18–20), suggesting that mother-child communication may play a greater role in family functioning in Chinese populations. A study shows that the quality of mother-child communication can negatively predict adolescent Internet addiction, but father-child communication has no significant predictive effect (21); this model may also apply to gambling disorders, as we speculate.

Impulsive personality, defined as acting without thinking, is a stable personality trait (22). Impulsive personality was positively correlated with distorted beliefs in patients with gambling disorder (23, 24); both were core processes related to gambling (25). Impulsivity is an innate trait, is a risk factor for addictive behaviors, such as GD (8, 25–27), and has conditions as a moderator. Additionally, as an interactive predictor, impulsivity has been found to moderate the influence of social factors on individuals (28, 29), so we believe that impulsivity also moderates the effects of family factors and mother-child communication in patients with gambling disorders, which are more closely related to individuals. According to Deng et al.'s model of adolescent Internet addiction, when risk factors (e.g., impulsivity) reach a certain level, the role of protective factors (e.g., parent-child communication) decreases (21).

The goal of this study was to examine the impact of parent-child communication on the general functioning of families and gambling-related beliefs in patients with gambling disorders, as well as the role of impulsivity in this model. Our hypotheses are as follows: (a) the general functioning of the family is positively correlated with parent-child communication; (b) the quality of parent-child communication is negatively correlated with gambling-related beliefs; (c) parent-child communication mediates the relationship between general family functioning and gambling-related beliefs; and (d) impulsivity moderates the indirect effect of family general functioning on gambling-related beliefs (through mother-child communication).

## 2. Method

### 2.1. Participants and procedure

A cross-sectional study was conducted. The participants consisted of 110 patients voluntarily seeking treatment for GD at the Behavioral Addictions Unit in Shanghai (China) or Shanghai Jiecheng Si Guoqi Gambling Abstinence Center from May 2021 to January 2022. After deleting four invalid samples and 11 samples that did not meet the DSM-5 criteria for GD, our sample now included 95 patients with GD; the mean age of the sample was 28.17 (SD = 4.49), and 95.80% were male. Inclusion criteria were as follows: (a) meeting the DSM-5 criteria for the diagnosis of GD, (b) age >18 years, and (c) willingness to participate in this study and provide informed consent. Exclusion criteria included (a) comorbid psychiatric or neurological disorders such as schizophrenia, (b) an intellectual disability, and (c) no parents' orphans when they were a child.

Participants who accepted to participate in the study were informed about the study's goal and ensured that their participation was voluntary and that they had the opportunity to withdraw at any time. The data were collected in the Behavioral Addictions Unit by well-trained data collectors. The study protocol and materials were approved by the ethics review committee of the Shanghai Mental Health Center, China. Clinical trial Registration Number: NCT03748875; Ethics Review Number: 2018KY-20. All subjects were informed about the study, and all provided written informed consent.

### 2.2. Measurements

#### 2.2.1. Family functioning

The Family Assessment Device (FAD) compiled by Epstein et al. was adopted to measure family functioning, which has good reliability and validity when applied to Chinese individuals (30, 31). The General Functioning 12-item subscale (GF12) is one of the dimensions and a shorter version of the FAD and gives a measure of the overall health/pathology of the family (32). Boterhoven de Haan et al. and Byles et al. have proven their confidence in the construct validity of the GF scale as a measure of family functioning (32, 33). The reliability of the GF scale was verified with an internal consistency of 0.86 (Cronbach's alpha).

#### 2.2.2. Parent-adolescent communication scale

The parent-Adolescent Communication Scale (PACS) developed by Aber-Huan et al. was adopted to measure communication between father/mother and child and applied to Chinese individuals with good reliability and validity (Cronbach's alpha = 0.89) (34, 35), and it was also widely used by adults in China (36, 37). In our study, Cronbach's alpha values were 0.84 and 0.82 on Parent-Adolescent Communication Scale for mother [PACS (M)] and father [PACS (F)]. The scale included two subscales with 10 items each: the open communication with parent subscale (OCPS) and the problems in communication with parent subscale (PCPS). In our study, Cronbach's alpha values were 0.80 and 0.81 for the OCPS and PCPS in the PASC (M) and 0.80 and 0.82 for the OCPS and PCPS in the PASC (F). The OCPS measures the positive aspects of communication between child and parent, and a higher score indicates a higher openness to communication. The PCPS measures the negative aspects of communication between child and parent, with a higher score indicating fewer communication problems. The total PACS score ranges from 20 to 80, with higher scores indicating better communication outcomes. PACS was used in our study to assess how well-subjects communicated with their parents during most of their lives, rather than specifically during childhood.

#### 2.2.3. Impulsivity

Barratt Impulsiveness Scale-version 11 (BIS-11) as a self-reported measure was used to assess impulsive traits (38, 39). Cronbach's alpha coefficient for the Chinese version was 0.76 (40). In our study, Cronbach's alpha was 0.75. The scale contains 30 items, and the higher the scores, the stronger the impulsivity.

#### 2.2.4. Gambling-related beliefs

The Gambling Attitude and Beliefs Survey (GABS) was established by Breen and Zuckerman in 1999 (41). Cronbach's alpha coefficient of the Chinese version was 0.76 (42), consisting of 35 items, and divided into three dimensions: cognition deviation, irrational beliefs, and active attitudes toward gambling. Higher scores on the scale indicate more distorted attitudes, beliefs, and perceptions toward gambling (41).

### 2.3. Statistical analysis

**Figure 1:**
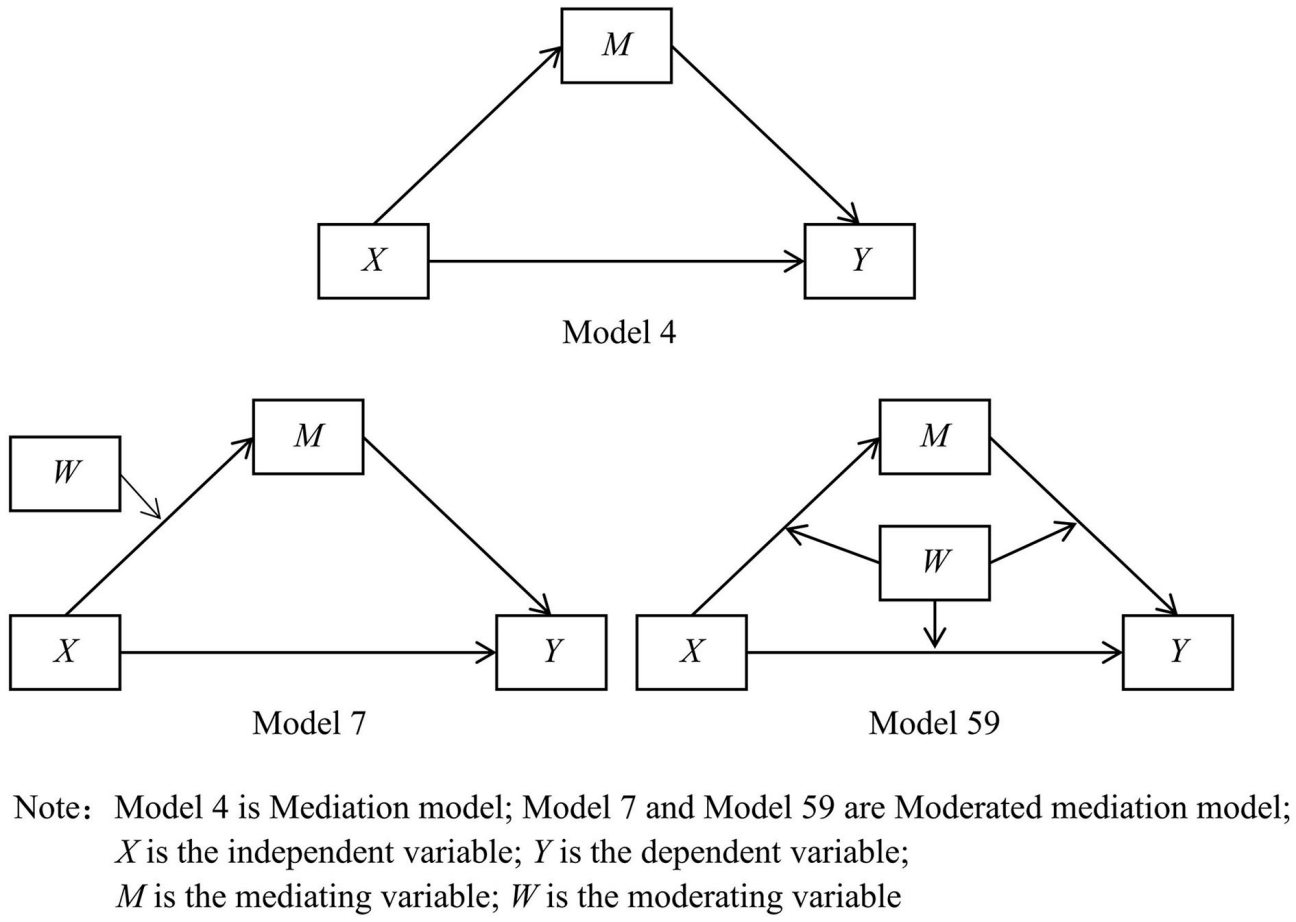
Model templates for PROCESS for SPSS.

We propose a partial correlation to investigate the relationship between the main important variables, such as FAD, PACS, BIS, and GABS, with age and gender as control variables in particular. The SPSS software (v.23.0 for Windows, IBM Corp., Armonk, New York, USA) was used for statistical analyses. A *p*-value of < 0.05 was considered to indicate statistical significance. The procedures of Baron and Kenny were used to examine the mediation hypotheses that we proposed earlier, conducting regression analyses with variables as follows: GABS as a dependent variable, GF12 as predictors separately, and PACS as mediators separately (43). According to the mediation procedure (44), the necessity of the significant direct effect of initial, independent variable *X* on outcome *Y* is no longer essential, and there may be a masking effect between variables *X* and *Y*. Therefore, the main effect may be weak or non-significant, and an indirect effect may exist (44–46). A moderated mediation model means that the independent variable influences the dependent variable through the mediation variable, and the mediation process is moderated by the moderating variable (43, 47). Furthermore, studies on the moderating effect test have shown that the correlation between ideal moderating variables and independent and dependent variables is not significant (48). We tested the Moderated Mediation Hypothesis 4 with BIS as a moderating variable. The Model 4, 7, and 59 for PROCESS for SPSS were used to assess the moderated mediation model (49, 50), see Figure 1. We estimated the significance of standardized coefficients with 5,000 bootstrap iterations. In this study, standard errors and confidence intervals for parameter estimations were obtained. Study results were considered statistically significant if the *p-*value was < 0.05 or the 95% confidence interval did not contain zero.

## 3. Results

### 3.1. Control and testing of common method biases

Based on procedural control of possible common method biases (such as reverse scoring of some items), the Harman single-factor test was used to conduct exploratory factor analysis on all items of the study variables. The results showed that there were 25 factors with Eigenvalues >1, and the explanatory rate of the first common factor was 14.19%, which was lower than the critical standard of 40%. Therefore, it could be inferred that there was no common method bias in this study.

### 3.2. Variable description statistics and correlation analysis

A total of 95 patients with GD were included in our study, with a mean age of 28.17 years (*SD* = 4.49), mean years of education of 14.63 years (*SD* = 2.53), mean duration of gambling of 14.63 years (*SD* = 2.53), 95.80% were male, and 85.30% gambled online, as shown in Table 1.

**Table 1:** The demographic characteristics of individuals with gambling disorder (*N* = 95).

**Characteristics**	**Type**	**Frequency**	**Mean ±standard deviation**
		**Percentage (%)**	
Sex	Male	91	95.80
	Female	4	4.20
Marriage	Unmarried	41	43.20
	Married	44	46.40
	Divorce	10	10.50
Job	Yes	66	69.50
	No	29	30.50
Way of GD	Online	81	85.30
	Underline	3	3.20
	Online and underline	11	11.60
Age (year)	28.17 ± 4.49
Education		14.63 ± 2.53	
Duration of GD		6.11 ± 4.00	

**Figure 2:**
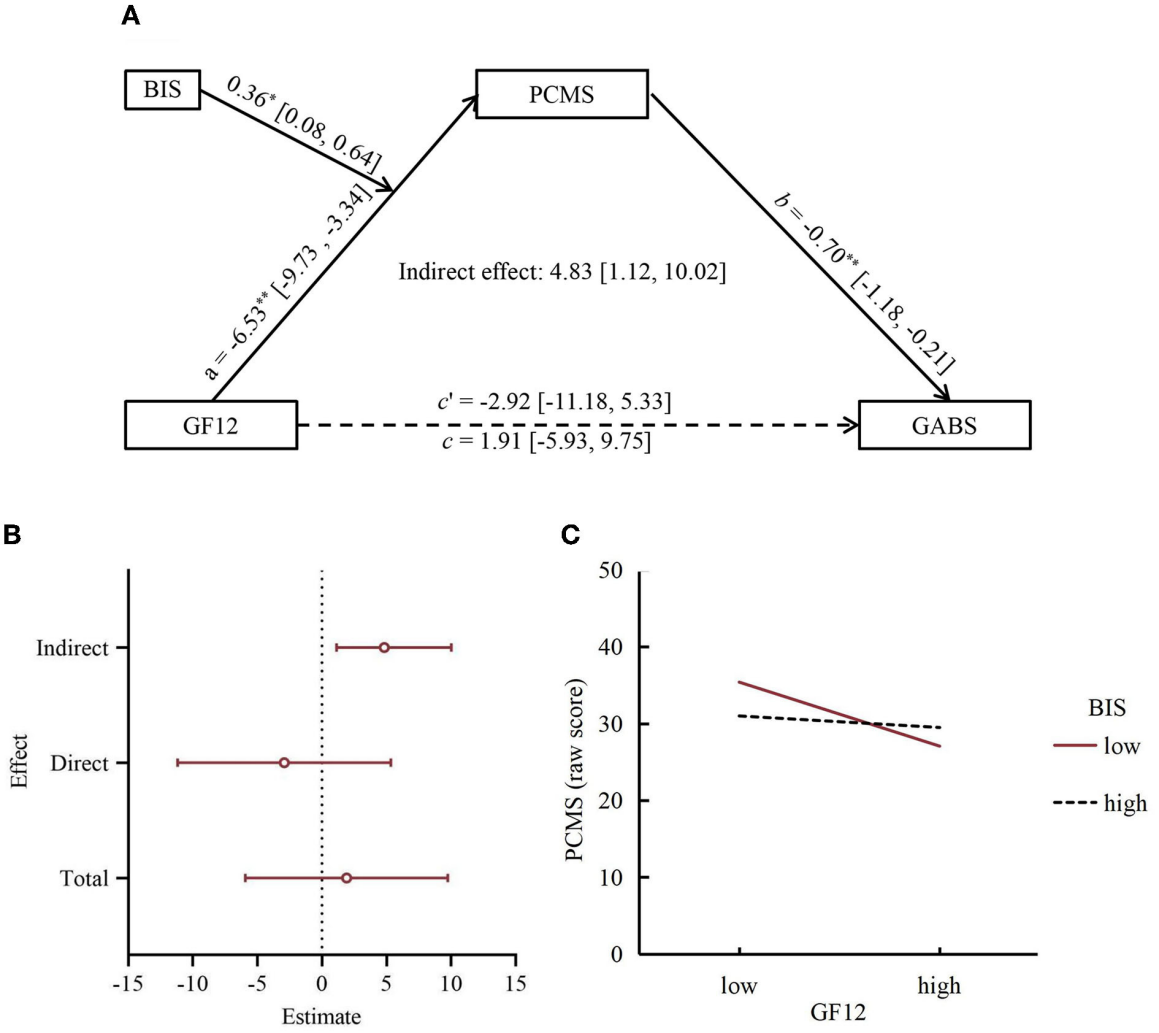
Illustrations of a modulated mediation model. **(A)** The moderated mediation model. Outside the brackets are the coefficients of the path, and inside the brackets are the lower limit and upper limit of a 95% confidence interval, which 0 is not included, it means that the prediction is significant. The 95% CI of the indirect effect coefficient does not contain 0, indicating that the indirect effect is significant. *p < 0.05, **p < 0.01. **(B)** Total, direct, and indirect effects of GF12 for GABS. The dot in the middle of the horizontal line represents estimates of the effect. The left and right endpoints of the horizontal line represent the lower and upper limits of the 95% confidence interval of the estimates of the effect, respectively, which 0 is not included, the effect is significant. **(C)** Moderating effects of impulsivity on GF12 and PCMS. low: Mean −1*SD*; high: Mean +1*SD, SD:* standard deviation.

We performed partial correlation analyses for GABS, BIS, GF12, PACS, and their subscales separately after inspecting their linearity with scatter plots and their normal distribution with the Kolmogorov–Smirnov test. The results showed that GABS was significantly correlated with the problems in communication with mother subscale (PCMS) but not correlated with the open communication with mother subscale (OCMS). There was no significant correlation between GABS and PACS (F), including its subscales (*P*-values > 0.05). As shown in Table 2, GF12 and PCMS in patients with gambling disorders were significantly negatively correlated, while PCMS was significantly negatively correlated with GABS. In addition, BIS was significantly positively correlated with GABS but not significantly correlated with GF12 and PCMS. We tested the parameters of some regression equations in the intermediate model with moderated front paths; specific results are shown in Tables 3, 4. The values of GF12, PCMS, and BIS were z-standardized to z-scores, and then z-scores of GF12 and PCMS were multiplied by the *Z*-scores of BIS as interaction points. Therefore, we adjusted the hypothesis model and obtained the final model as shown in Figure 2A. The specific verification process is as follows.

**Table 2:** Descriptive statistics and correlation coefficients (*r*'s) among variables (*N* = 95).

**Item**	***M* (SD, skewness, kurtosis)**	**GABS**	**GF12**	**BIS**	**PACS(M)**	**PCMS**	**OCMS**	**PACS(F)**	**PCFS**
GABS	106.55 (15.49, 0.03, 0.77)								
GF12	2.30 (0.48, −0.86, 2.84)	0.24^**^							
BIS	51.74 (11.17, −0.21, −0.41)	0.29^**^	0.15						
PACS(M)	55.74 (12.71, −0.02, 0.06)	−0.28^**^	−0.62^**^	−0.13					
PCMS	21.59 (6.50, 0.10, 0.52)	−0.29^**^	−0.41^**^	−0.07	0.81^**^				
OCMS	34.16 (8.01, −0.37, −0.13)	−0.17	−0.63^**^	−0.02	0.91^**^	0.52^**^			
PACS(F)	60.22 (7.66, −1.12, 5.76)	0.13	−0.24^**^	0.04	0.02	−0.21^*^	0.18^*^		
PCFS	29.66 (6.81, −0.23, 0.72)	0.12	0.33^**^	0.17	−0.35^**^	−0.46^**^	−0.23^*^	0.32^**^	
OCFS	30.56 (7.97, 0.17, 0.47)	−0.16	−0.56^**^	−0.13	0.36^**^	0.22^*^	0.39^**^	0.56^**^	−0.51^**^

M, mean value; SD, standard deviation; ^*^p < 0.05; ^**^p < 0.01, same below.

GBAS, gambling Attitude and Belief Scale; GF12, general functioning 12-items of family assessment device; PACS (M), Parent-Adolescent Communication Scale (for mother); PCMS, the problems in communication with mother subscale; OCMS, the open communication with mother subscale; PACS (F), Parent-Adolescent Communication Scale (for father); PCFS, the problems in communication with father subscale; OCFS, the open communication with father subscale; BIS, Barratt Impulsiveness Scale.

**Table 3:** Mediation model with PCMS as the mediator variable.

**Variable**	**Equation 1 (dependent variable: PCMS)**				**Equation 2 (dependent variable: GABS)**				**Equation 3 (total, direct, and indirect effect)**		
	β	**SE**	***t***	**95% CI**	β	**SE**	***t***	**95% CI**	β	**SE**	**95% CI**
GF12	−6.95^***^	1.65	−4.22	(−10.22, −3.68)	−2.92	4.15	−0.70	(−11.18, 5.33)			
PCMS					−0.70^**^	0.24	−2.88	(−1.18, −0.21)			
Gender	1.20	3.18	0.38	(−5.11, 7.51)	−2.05	7.33	−0.28	(−16.61, 12.52)			
Age	0.37^*^	0.14	2.62	(0.09, 0.65)	0.40	0.34	1.18	(−0.27, 1.07)			
Total effect									1.91	3.95	(−5.93, 9.75)
Direct effect									−2.92	4.15	(−11.18, 5.33)
Indirect effect									4.83	2.27	(1.12, 10.02)
*R*^2^ (%)	21.18				8.90				0.53		
F	8.15				2.20				0.16		

The 95% interval of all predictive variables was obtained by bootstrapping.

SE, standard error; CI, confidence interval.

^*^p < 0.05. ^**^p < 0.01. ^***^p < 0.001.

95% Bootstrap CI does not contain 0: the findings were considered statistically significant.

**Table 4:** Model of general functioning of family relationships to gambling-related beliefs.

**Variable**	**Equation 1 (Modle 59) (dependent variable: GABS)**				**Equation 2 (Modle 7) (dependent variable: PCMS)**				**Equation 2 (Modle 7) (dependent variable: GABS)**			
	β	**SE**	***t***	**95% CI**	β	**SE**	***t***	**95% CI**	β	**SE**	***t***	**95% CI**
GF12	−3.13	4.17	−0.75	(−11.43, 5.16)	−6.53^***^	1.61	−4.06	(−9.73, −3.34)	−2.92	4.15	−0.70	(−11.18, 5.33)
BIS	0.36^**^	0.13	2.70	(0.10, 0.63)	−0.04	0.06	−0.63	(−0.15, 0.08)				
GF12 × BIS	0.24	0.38	0.54	(−0.55, 0.97)	0.36^*^	0.14	2.58	(0.08, 0.64)				
PCMS × BIS	−0.02	0.02	−0.85	(−0.06, 0.02)								
PCMS	−0.71^**^	0.25	−2.88	(−1.20, −0.22)					−0.70	0.24	−2.88	(−1.18, −0.21)
Index of MM									−0.25	0.14		(−0.60, −0.04)
*R*^2^ (%)	16.98^**^				27.72^***^				8.90			
F	2.54				6.83				2.20			

Equation 1: Model 59 in the Process program developed by Hayes was used for testing.

Equation 2: Model 7 in the Process program developed by Hayes was used for testing.

The 95% interval of all predictive variables was obtained by bootstrapping.

SE, standard error; CI, confidence interval; MM, moderated mediation.

^*^p < 0.05. ^**^p < 0.01. ^***^p < 0.001.

95% Bootstrap CI does not contain 0: the findings were considered statistically significant.

### 3.3. The mediation effect of PCMS between GF12 and GABS

As shown in Table 3, model 4 in the SPSS process program was used to test and analyze the mediation effect of PCMS between GF12 and GABS by the bootstrapping method. First, the total effect coefficient *c* (β = 1.91) from GF12 to GABS was tested, and the 95% bootstrap CI is (−5.93, 9.75), which overlaps 0, indicating that GF12′s total prediction effect on GABS is not significant. It is considered that there may be a masking effect between GF12 and GABS. Next, the pre-path and post-path coefficients *a* and *b* are tested: *a* (β = −6.95, *t* = −4.22, *p* < 0.01) and *b* (β = 0.70, *t* = 2.88, *p* < 0.01), indicating that GF12 has a significant negative prediction effect on PCMS, and PCMS has a significant negative prediction effect on GABS. Then, the direct effect coefficient *c*′ (β = −2.92, *t* = −0.70, *p* > 0.05) indicates that GF12 has no significant direct prediction effect on GABS, so it can be inferred that PCMS has a complete mediation effect between GF12 and GABS. Finally, the bootstrap method was used to test the mediating effect of PCMS between GF12 and GABS. The results showed the indirect effect coefficient β = 4.83 [95% CI (1.12, 10.02)], indicating that PCMS had a significant mediating effect (see Figures 2A, B). We also tested the Father-Child Communication Scale and its subscales as mediating variables, and the results showed that their 95% confidence intervals of effect values overlapped with 0, indicating that they had no mediating effect between GF12 and GABS (data not shown here for simple reasons).

### 3.4. Moderating effect of impulsivity on PCMS

Existing studies on the moderating effect test have shown that the correlation between ideal moderating variables and independent and dependent variables is not high (48). Table 2 correlation analysis results showed that impulsivity was not significantly correlated with GF12 and PCMS except for GABS. Therefore, impulsivity met the requirements of the moderating effect test.

First, Model 59 in the process program developed by Hayes was used to test the moderated mediation effect. The results showed that only the product term of GF12 and BIS had a significant predictive effect on PCMS after adding the moderating variable BIS into the model (β = 0.36, *t* = 2.58, *p* < 0.05), indicating that BIS only plays a moderating role in GF12′s prediction of PCMS (see Table 4, Equation 1). This result was further verified by using Model 7 in the process program (see Table 4, Equation 2). Therefore, GF12, PCMS, BIS, and GABS constitute a moderated mediation model, and the moderating variables regulate only the front path of the mediation process. To further investigate the nature of the moderating effect, a simple slope analysis was conducted. The results showed that in terms of the effect of GF12 on PCMS, the score of PCMS decreased with the increase in GF12 score, and compared with high BIS, PCMS decreased more obviously at low BIS (see Figure 2C).

Moreover, as shown in Table 5, the indirect positive effect between GF12 and GABS via PCMS was stronger for a child with a low BIS [Eff1 = 7.37, SE = 3.27, 95% CI (1.65, 14.61)] than for a child with a high BIS [Eff3 = 1.72, SE = 1.94, 95% CI (−1.64, 6.15)], and the contrast between moderating mediating effects of impulsivity at different levels was significant [Eff3–Eff1 = −5.65, SE = 3.15, 95% CI (−13.03, −0.74)].

**Table 5:** Mediating effects at different levels of impulsivity.

	**Impulsivity**	**Effect**	**BootSE**	**95% CI**
Moderating mediating effect	Eff 1 (M−1SD)	7.37	3.27	(1.65, 14.61)
	Eff 2 (M)	4.54	2.18	(0.93, 9.57)
	Eff 3 (M−1SD)	1.72	1.94	(−1.64, 6.15)
Pairwise contrasts between moderating mediating effects	Eff 2 – Eff 1	−2.83	1.58	(−6.52, −0.37)
	Eff 3 – Eff 1	−5.65	3.15	(−13.03, −0.74)
	Eff 3 – Eff 2	–−2.83	1.58	(−6.52, −0.37)

95% Bootstrap CI does not contain 0: the findings were considered statistically significant.

## 4. Discussion

We used a moderated mediation model to investigate the effects of parent-child communication on family functioning and gambling-related beliefs in patients with gambling disorder and the role of impulsivity in this model. Our model shows that (1) the quality of the general functioning of the family is positively correlated with the quality of mother-child communication; (2) the quality of mother-child communication is negatively correlated with distorted gambling-related beliefs; (3) the problems in communication with the mother mediate the relationship between the general functioning of the family and gambling-related beliefs; and (4) impulsivity moderates the mediating effect through the pattern between the general functioning of the family and the problems in communication with the mother. These results suggest that our hypothesis holds only when mother-child communication problems, rather than father-child communication problems, are used as mediating variables. These results also indicate the complex interaction relationship between personal traits and family environment in behavior addiction, which will be discussed in the following sections.

### 4.1. The mediating role of the problems in communication with the mother

The mediating model of this study suggests that the relationship between dysfunctional family functioning and gambling attitudes operates through poor communication with the mother, which focuses on caution and selectivity in the exchange of information and negative styles of interaction. The higher the level of family functioning, the fewer the conflicts between family members; the more harmonious the family relationship and communication mode, the fewer problems in communication with the mother, which is consistent with previous research views (18). We found that the overall level of mother-child communication was high compared to that of father-child communication. Problems in communication with the mother can significantly predict an adult's gambling behavior, but the predictive effect of father-child communication was not significant, showing that mother-child communication played a more significant protective role for adults. This may be because adults with fewer problems in communication with their mothers (higher PCMS scores) may be more willing to communicate with their mothers when facing gambling problems so that they can easily get more support and improve their gambling-related beliefs. These results are similar to those of Deng et al.'s study on the interaction of impulsive personality and parent-child communication on adolescent Internet addiction (21). Family members, especially mothers, may recognize and respond to an adult's gambling problem earlier. In conclusion, the better the family functioning, the fewer the communication problems between mother and adult child, the healthier the gambling-related beliefs, and their gambling behavior will be greatly reduced.

### 4.2. The moderating effect of impulsivity on the general functioning of the family, problems in communication with the mother, and gambling-related beliefs

Impulsivity moderated the pathway from the general functioning of the family to problems in communication with the mother. In other words, the severity of problems in communication with the mother in patients with low impulsivity gambling disorder was more likely to be affected by the general functioning of the family than those with high impulsivity gambling disorder, and their gambling-related beliefs were also more likely to be indirectly affected. This verifies the *condition model of influence*, that is, the influence of family factors on individuals is moderated by temperament or personality factors. We think that the risk of aggravating gambling disorder brought on by high impulsivity in patients is offset by the protective effect of family function and parent-child communication, which makes it difficult for them to get timely persuasion and correction of their distorted gambling-related beliefs, and they are more likely to fall into pathological gambling. This result, to some extent, supports the “protective-reactive model” (51) modified in the theory of the “organism-environment interaction model” (4), that is, only when risk factors are at low levels, protective factors will play a greater role. When the risk factors reach a certain level, the protective factors will reduce their role and even change the direction of action.

In addition, impulsivity has a direct positive predictive effect on gambling-related beliefs. This is consistent with the results of other studies (52–54).

### 4.3. Practical implications

From a clinical perspective, it can be assumed that the results of this study may provide information for potential therapeutic targets in the future. The effect of impulsivity suggests that psychotherapy for impulsivity may reduce the damaging effect of risk factors. Specifically, when individuals more actively seek treatment for impulsivity problems, such as contingency management (CM), with or without cognitive behavioral therapy (CBT) to reduce impulsivity will lead to better outcomes (55). Furthermore, therapy for improving family functioning can play a greater protective role, improve problems in communication with the mother, and further correct patients' gambling-related beliefs. As a result, family and individual psychotherapy and assessment work for people with gambling disorders may need to be adapted in a more targeted manner to address impulsivity and the problems in communication with the mother, with a special emphasis on the latter, though this is currently speculative based on recent findings. Specific supplementary therapy aimed at impulsivity linked to GD severity may help patients achieve better treatment outcomes. It has been observed that the use of therapeutic video games as an additional therapeutic tool can treat difficulties in emotional regulation and impulsivity (56, 57).

### 4.4. Limitations and future research

Of course, our results should also take into account the limitations of the study. First, the small sample size and obvious gender bias may affect the generalization of the research conclusions. Second, as a cross-study, we failed to examine the characteristics of changes in gambling-related beliefs of patients with gambling disorder at different developmental stages and the effects of family functioning, mother-child communication, and impulsivity on gambling-related beliefs. These studies should be examined in future studies. Third, this study failed to investigate the influence of other family background factors on GABs, such as the family structure of the subjects and the one-child or non-one-child family. Fourth, there are no comparisons or discussions with widely known baselines in the field. Despite these limitations, the study provides important data to support and refine the theoretical model proposed for specific forms of GABs and suggests important avenues for intervention strategies to improve attitudes and beliefs about gambling and thereby reduce gambling behavior.

## Data availability statement

The raw data supporting the conclusions of this article will be made available by the authors, without undue reservation.

## Ethics statement

The studies involving human participants were reviewed and approved by the Ethics Review Committee of Shanghai Mental Health Center, China. The patients/participants provided their written informed consent to participate in this study.

## Author contributions

DZ: writing—original draft preparation, writing, reviewing and editing, and formal analysis. SZ: writing—original draft preparation, data curation, and formal analysis. HZ: conceptualization, writing, reviewing, editing, and project administration. LG and ZL: formal analysis. JZ, ZD, and PD: investigation. MZ: supervision. JD: conceptualization and supervision. All authors contributed to the article and approved the submitted version.
